# A Balanced Substrate Integrated Waveguide Phase Shifter with Wideband Common-Mode Suppression

**DOI:** 10.3390/mi14020285

**Published:** 2023-01-22

**Authors:** Wei Zhang, Jin Shi, Gangxiong Wu, Longlong Lin, Kai Xu

**Affiliations:** 1 School of Information Science and Technology, Nantong University, Nantong 226019, China; 2 Research Center for Intelligent Information Technology, Nantong University, Nantong 226019, China; 3 Nantong Key Laboratory of Advanced Microwave Technology, Nantong University, Nantong 226019, China; 4 Zhongtian Radio Frequency Cable Co., Ltd., Nantong 226010, China

**Keywords:** balanced phase shifter, common-mode suppression, substrate integrated waveguide, wideband

## Abstract

In this paper, a slotted substrate integrated waveguide (SIW) is used to create a balanced phase shifter with wideband common-mode (CM) suppression. Differential-mode (DM) impedance matching and CM suppression are achieved by utilizing the fact that TE_20_ mode and TE_10_ (TE_30_) mode can only transmit DM signals and CM signals, respectively, and by increasing the bandwidth for CM suppression via slots. Furthermore, a wideband phase shift with low phase deviation can be obtained due to the phase slop counteract between the slot and the delay line. Compared with the state-of-the-art, the proposed one has the advantages of wideband CM suppression, wide phase shift range, and a simple and easy-to-make structure. Five prototypes covering the frequency of 3.5 GHz are designed with the relative operating bandwidth for 45° ± 2° (90° ± 4.5°, 135° ± 6°, and 180° ± 8°) phase shifter of 20% (20%, 20%, and 20%), with the minimum insertion loss of 0.41 dB (0.5 dB, 0.58 dB, and 0.59 dB), with the minimum return loss greater than 15 dB, and with the relative bandwidth for 15-dB CM suppression of 59% (59%, 58%, and 57%).

## 1. Introduction

Phase shifters play crucial roles in wireless communication systems, such as radar, phase modulators, beamforming networks, and phased array antenna systems, because they can provide the required phase shifts [1,2,3]. The increasing popularity of balanced phase shifters is due to their high immunity to electromagnetic interference and their simple connection to other balanced circuits or antennas. In addition to the advantages of the balanced microstrip phase shifter, the balanced substrate integrated waveguide (SIW) phase shifter has high-quality factors, a high power handling capacity, and low loss. Consequently, the balanced SIW phase shifter will be in high demand in modern communication systems. Balanced SIW phase shifter design challenges include wideband CM suppression while preserving wideband DM phase shift, low in-band phase deviation, and low insertion loss.

Currently, a few balanced phase shifters have been reported [4,5,6,7,8,9,10,11], and all of them are microstrip designs. Several approaches have been used to design microstrip balanced phase shifters, including coupled lines with loaded transmission lines [4,5,6], two-stage branch line structure [7], cascaded three microstrip lines and two coupled lines [8], T-shaped multi-mode resonator with intermediate cascaded stubs [9], two three-end folded coupled lines with three half-wavelength microstrip lines [10], LC-based microstrip differential line with periodic T-shaped stubs [11], etc. Among them, different features have been achieved, such as compact size in [4,6], wideband CM suppression in [8], large phase shift range in [7,9], fusing filtering function in [5,10], and tunable phase shift in [11].

To our knowledge, no balanced SIW phase shifter has been reported till now. Various single-ended SIW phase shifters have been reported [12,13,14,15,16,17,18,19,20,21,22,23,24], and their design methods can be categorized as inserted metallic posts [12,13,14], buried PIN diodes [15], embedded omega particles [16], CSRR-loaded SIW [17], air-filled SIW loaded with dielectric slab [18], loaded rod-shaped artificial dielectric slabs [19], slow-wave structures [20], slotted SIW [21,22], varactor-loaded slotted SIW [23], transverse slot coupling [24], and so on. Among the aforementioned SIW phase shifters, some designs pursue equal lengths of the reference and main lines ([12,13,15,17]), some designs obtain wide operating bandwidths ([14,16,19,20,21]), some designs attain low insertion loss ([18,22]), and some designs realize tunable phase shift ([23,24]).

This paper constructs a balanced SIW phase shifter with wideband CM suppression by combining slotted SIW and microstrip delay lines. It is possible to simultaneously achieve wideband DM impedance matching, wideband phase shift with low in-band phase deviation, and wideband CM suppression. The equivalent circuit of the centred slot on the SIW and the surface current distributions of the SIW with/without slots are analysed to introduce the functions of the slots on the top surface of the SIW. The parametric study and the design procedure are provided to guide the practical design. Five prototypes are fabricated to verify the theoretical prediction.

## 2. Proposed Balanced SIW Phase Shifter

### 2.1. Structure

**Figure 1:**
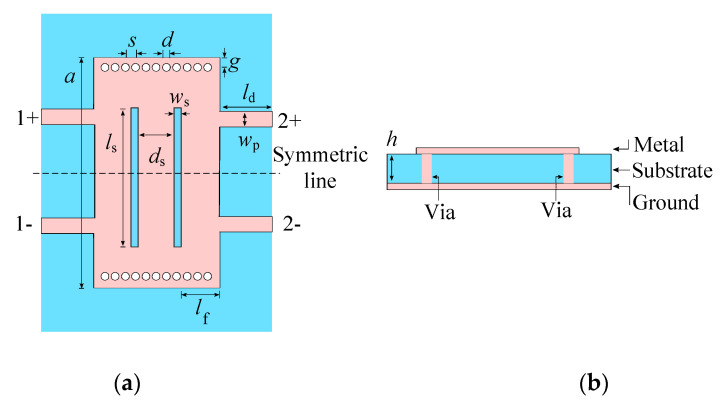
Structure of the proposed balanced SIW phase shifter. (**a**) Top view. (**b**) Cross-sectional view.

The proposed balanced SIW phase shifter is constructed by etching two symmetrical transverse slots into the top surface of SIW, as shown in Figure 1. Two pairs of symmetric microstrip lines form the two balanced ports, namely 1+ and 1−, 2+ and 2−, and the microstrip feed lines, which serve as delay lines. The reference and main lines employ the same circuit model on the RO4003C substrate (the dielectric constant of 3.38, thickness of 0.813 mm, and loss tangent of 0.0027). The lengths of the delay lines and the slot parameters distinguish the reference line from the main line.

An SIW is equivalent to a conventional dielectric-filled metallic rectangular waveguide, and the equivalent relationship can be described as [25]:

(1) $$w_{\mathrm{eff}}=a-\frac{d}{0.95s}$$

where *w*_eff_ is the equivalent width of the SIW compared with its conventional rectangular counterpart, *a* is the SIW width, and *d* and *s* are the diameter of metallic vias and the space between adjacent metallic vias, respectively. The dimensions of *a*, *d,* and *s* should follow the rules given in [26].

To achieve the transmission of DM signals and the suppression of CM signals at the centre frequency (*f*_0_), the distance between ports 1+(2+) and 1−(2−) is set to half the equivalent width of the SIW. In addition, the relationship between *f*_0_ and the cut-off frequencies of TE_20_ mode and TE_30_ mode must be satisfied as:

(2) $$f_{c({\mathrm{TE}}_{20})}<f_{0}<f_{c({\mathrm{TE}}_{30})}$$

Thus, *w*_eff_ can also be expressed as:

(3) $$\frac{c_{0}}{f_{0}\sqrt{\varepsilon_{r}}}<w_{\mathrm{eff}}<\frac{3c_{0}}{2f_{0}\sqrt{\varepsilon_{r}}}$$

Therefore, *w*_eff_ can be obtained according to *f*_0_. Conversely, *f*_0_ can be controlled by *w*_eff_.

### 2.2. DM Analysis

**Figure 2:**
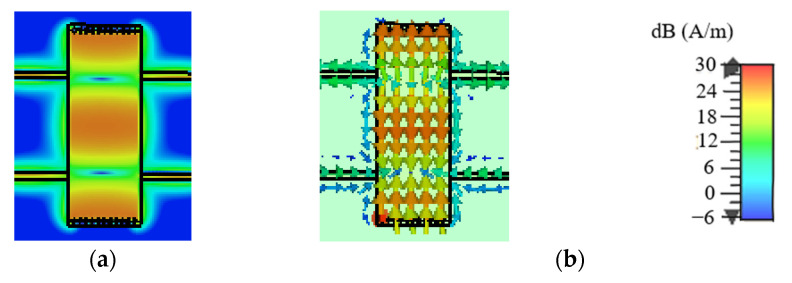
The surface current distributions on the SIW without slots for DM operating at *f*_0_. (**a**) The uniform amplitude distributions. (**b**) The vector distributions.

**Figure 3:**
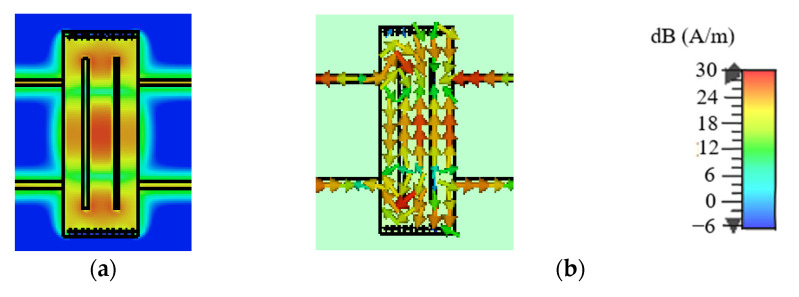
The surface current distributions on the SIW with two slots for DM operating at *f*_0_. (**a**) The uniform amplitude distributions. (**b**) The vector distributions.

**Figure 4:**
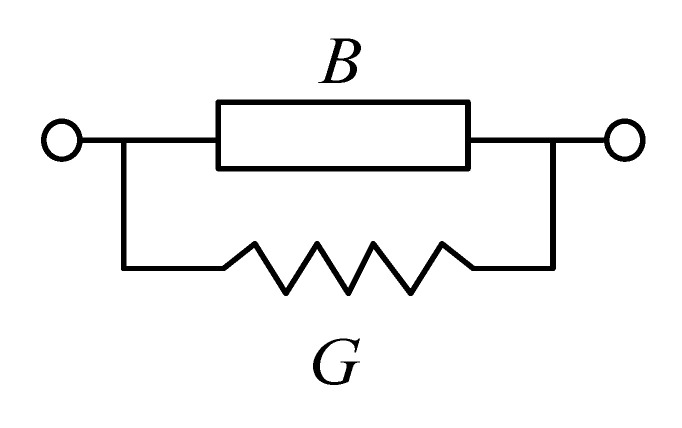
The equivalent circuit of the centred slot on the top surface of SIW.

Figure 2 and Figure 3 illustrate the uniform amplitude distributions and vector distributions of the surface current on the SIW with/without slots for DM operating at *f*_0_ to determine the effects of slots on DM properties. Figure 2 and Figure 3 demonstrate that the TE_20_ mode will be excited when the proposed design is operating at the DM. In addition, Figure 2 reveals that the surface current on the SIW without slots is distributed uniformly in the transmission direction, whereas the surface current on the SIW with two slots in Figure 3 is disturbed by the slots. It can be observed from Figure 3 that the transmission paths of the surface current on the upper and lower edges of the SIW are changed, and the surface current avoids the slots when transmitting forward. In addition, the surface current in the middle position of the SIW is transmitted through the slots’ coupling. Therefore, the slots will change the transmission mechanism and the distributions of the surface current on the SIW; this is because a centred slot on the top surface of SIW can be equivalent to a circuit with a susceptance (*B*) and a conductance (*G*) in parallel [27], as shown in Figure 4, where *B* and *G* can be written as Equations (4)–(13).

(4) $$G=Y_{0}\frac{32\lambda_{g}w_{\mathrm{eff}}b}{3\lambda^{3}\pi }[1-0.374(\frac{l_{s}}{\lambda }{)}^{2}+0.13(\frac{l_{s}}{\lambda }{)}^{4}][\frac{\pi }{4}\frac{1-{\left(l_{s}/w_{\mathrm{eff}}\right)}^{2}}{\cos(\pi l_{s}/2w_{\mathrm{eff}})}{]}^{2}$$

(5) $$B=Y_{0}\left\{\frac{1}{2}\frac{B_{t}}{Y_{0}}+\frac{B_{rj}}{Y_{0}}\frac{1}{{n_{j}}^{2}}+\frac{2b}{\lambda_{g}}\left[\ln2+\frac{\pi w_{s}}{6b}+\frac{3}{2}(\frac{b}{\lambda_{g}}{)}^{2}\right]\right\}$$

where *b* is the thickness of the substrate, and

(6) $$B_{t}=Y_{0}\left\{\frac{4b}{\lambda_{g}}[\ln\csc\frac{\pi w_{s}}{2b}+\frac{1}{2}(\frac{b}{\lambda_{g}}{)}^{2}\cos^{4}(\frac{\pi w_{s}}{2b})]-\frac{4b}{\lambda_{g}}(\frac{\lambda_{g}}{\lambda_{g3}}{)}^{2}[1+(\frac{\pi w_{s}}{2\lambda_{g3}}{)}^{2}][\frac{\left(1-(\frac{l_{s}}{w_{\mathrm{eff}}}{)}^{2})\cos(\frac{3\pi l_{s}}{2w_{\mathrm{eff}}}\right)}{\left(1-9(\frac{l_{s}}{w_{\mathrm{eff}}}{)}^{2})\cos(\frac{\pi l_{s}}{2w_{\mathrm{eff}}}\right)}{]}^{2}\ln(\frac{4\lambda_{g3}}{1.781\pi w_{s}})\right\}$$

(7) $$\lambda_{g3}=\frac{\lambda }{\sqrt{1-{\left(\frac{3\lambda }{2w_{\mathrm{eff}}}\right)}^{2}}}$$

(8) $$B_{rj}=Y_{0}\frac{2w_{s}\lambda_{g}}{\lambda^{2}}\left\{\left(\frac{\kappa \prime }{k}{)}^{2}[C+1.5-\ln\frac{\gamma \left|\kappa \prime \right|w_{s}}{2}]+\frac{\sin(kl_{s})}{kl_{s}}+[1+(\frac{\lambda }{2l_{s}}{)}^{2}](S\_)-\frac{2w_{s}}{3l_{s}}(\frac{\lambda }{2l_{s}}{)}^{2}\right)\right\}$$

(9) $$C=\frac{C_{i}(kl_{s}+\pi )+C_{i}\left|kl_{s}-\pi \right|}{2}$$

(10) $$S\_=\frac{S_{i}(kl_{s}+\pi )-S_{i}(kl_{s}-\pi )}{2\pi }$$

(11) $$S_{i}(x)=\int_{0}^{x}\frac{\sin t}{t}dt$$

(12) $$C_{i}(x)=\int_{0}^{x}\frac{\cos t}{t}dt$$

(13) $$\frac{1}{{n_{j}}^{2}}=\frac{w_{\mathrm{eff}}b}{l_{s}w_{s}}[\frac{\pi }{4}\frac{1-{\left(l_{s}/w_{\mathrm{eff}}\right)}^{2}}{{\left(\pi l_{s}/2w_{\mathrm{eff}}\right)}^{2}}{]}^{2}$$

**Figure 5:**
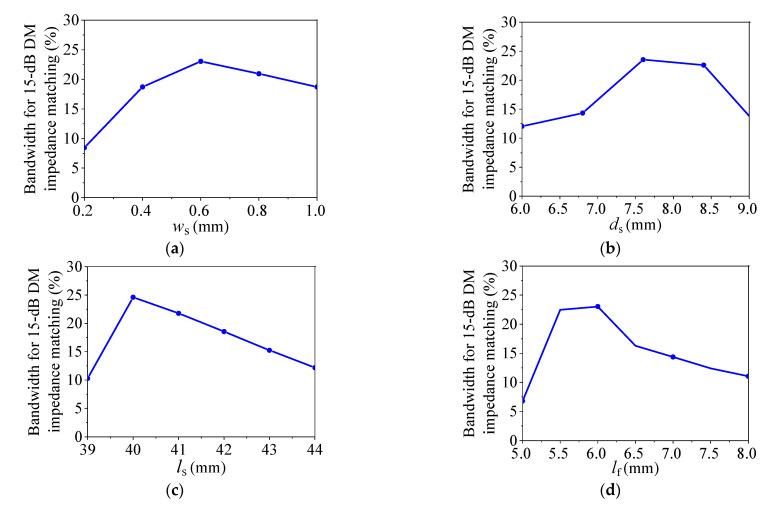
The simulated bandwidth for 15-dB DM impedance matching of the proposed balanced SIW phase shifter with different *w*_s_, *d*_s_, *l*_s_, and *l*_f_. (**a**) With different *w*_s_. (**b**) With different *d*_s_. (**c**) With different *l*_s_. (**d**) With different *l*_f_.

**Figure 6:**
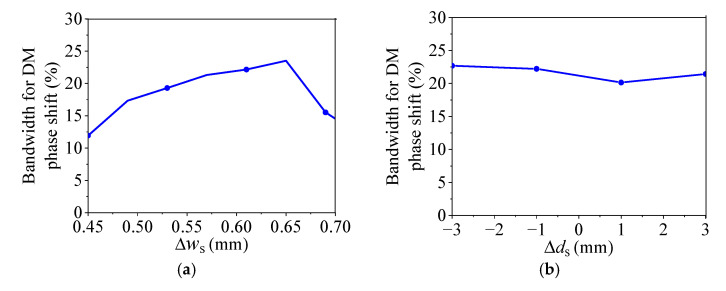

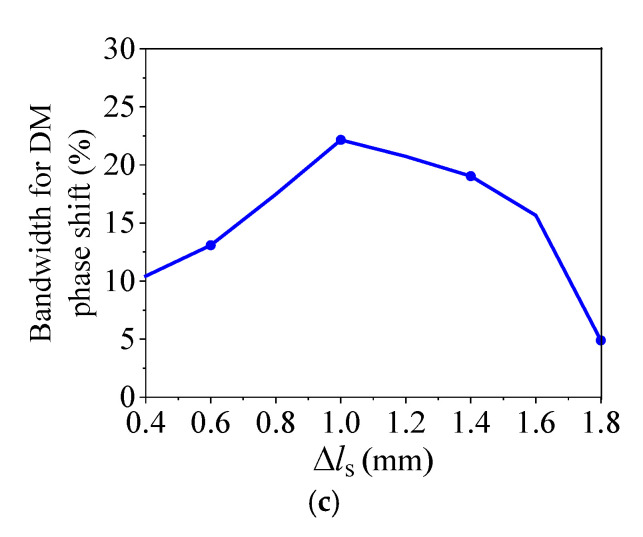
The simulated bandwidth for DM phase shift with ±5% phase deviation of the proposed balanced SIW phase shifter with different Δ*d*_s_, Δ*w*_s_, and Δ*l*_s_. (**a**) With different Δ*d*_s_. (**b**) With different Δ*w*_s_. (**c**) With different Δ*l*_s_.

According to Equations (4)–(13), the values of *B* and *G* are mainly determined by the parameters of the SIW and the slots. Thus, to further study how the parameters of slots on the SIW affect the properties of DM impedance matching and DM phase shift of the proposed balanced SIW phase shifter, Figure 5 provides the variations of the simulated bandwidth for the 15-dB DM impedance matching with different *w*_s_, *d*_s_, *l*_s_, and *l*_f_. Figure 6 shows the variations of the simulated bandwidth for the DM phase shift with ±5% phase deviation for different Δ*d*_s_ (*d*_sr_ − *d*_sm_), Δ*w*_s_ (*w*_sr_ − *w*_sm_), and Δ*l*_s_ (*l*_sr_ − *l*_sm_), where the subscripts *r* and *m* of *d*_sr_, *d*_sm_, *w*_sr_, *w*_sm_, *l*_sr_, and *l*_sm_ indicate the reference and main lines, respectively.

It can be observed from Figure 5 that the bandwidth for 15-dB DM impedance matching increases first and then decreases when increasing *w*_s_, *d*_s_, *l*_s_, or *l*_f_. Therefore, the wideband DM impedance matching can be achieved by selecting the proper parameters of *w*_s_, *d*_s_, *l*_s_, or *l*_f_. Figure 6a,c show that the bandwidth of DM phase shift with ±5% phase deviation increases at first and then decreases as increasing Δ*w*_s_ or Δ*l*_s_. Figure 6b demonstrates that Δ*d*_s_ has a negligible effect on the bandwidth of the DM phase shift with ±5% phase deviation. Thus, the wideband DM phase shift with ±5% phase deviation can be obtained by selecting the parameters of Δ*w*_s_ or Δ*l*_s_ appropriately.

### 2.3. CM Analysis 

**Figure 7:**
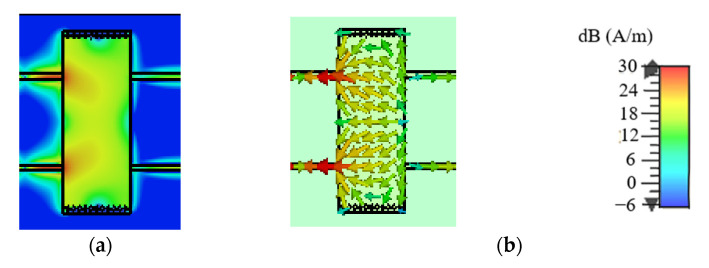
The surface current distributions on the SIW without slots for CM operating at *f*_0_. (**a**) The uniform amplitude distributions. (**b**) The vector distributions.

**Figure 8:**
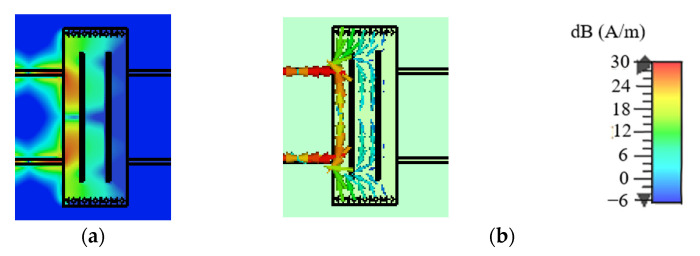
The surface current distributions on the SIW with two slots for CM operating at *f*_0_. (**a**) The uniform amplitude distributions. (**b**) The vector distributions.

Similarly, Figure 7 and Figure 8 depict the uniform amplitude and vector distributions of the surface current on the SIW with/without slots for CM operating at *f*_0_. It can be observed from Figure 7 that a small portion of the surface current on the SIW without slots can be transmitted, while the remainder will be substantially suppressed. Figure 8 demonstrates that on an SIW with two slots, almost no surface current can be transmitted, indicating the current is effectively prevented by the slots. Therefore, the CM suppression will be further enhanced by the slots.

Moreover, according to Equation (2), the frequency space between TE_10_ and TE_30_ modes would be wider than the DM operating frequency range if *f*_0_ were fixed. Therefore, it is possible to achieve CM suppression with a bandwidth that completely encompasses the DM operating bandwidth.

**Figure 9:**
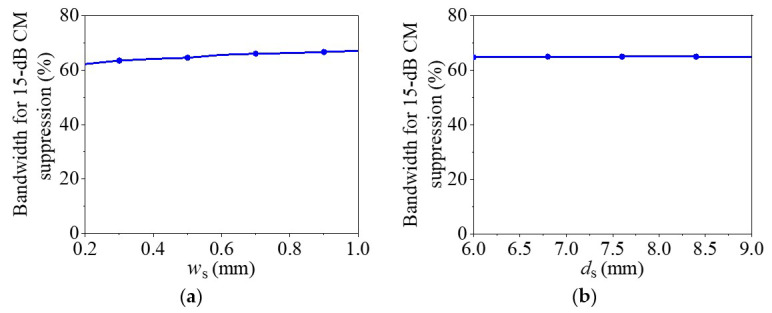

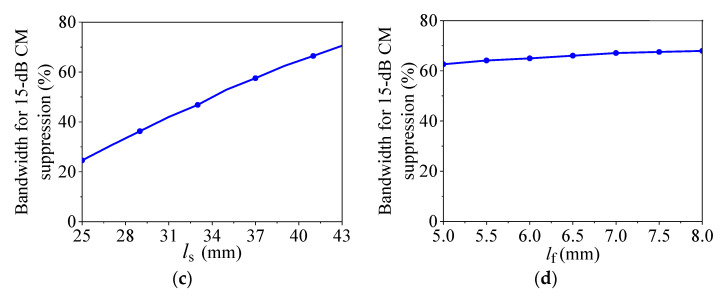
The simulated bandwidth for 15-dB CM suppression of the proposed balanced SIW phase shifter with different *w*_s_, *d*_s_, *l*_s_, and *l*_f_. (**a**) With different *w*_s_. (**b**) With different *d*_s_. (**c**) With different *l*_s_. (**d**) With different *l*_f_.

Figure 9 displays the simulated bandwidth for 15-dB CM suppression with varying *w*_s_, *d*_s_, *l*_s_, and *l*_f_ to illustrate how the slot parameters influence the CM suppression of the proposed balanced SIW phase shifter. It can be observed from Figure 9a,d that the bandwidth for CM suppression increases slightly with increasing *w*_s_ or *l*_f_. Figure 9b shows that the bandwidth for CM suppression is nearly unchanged with varying *d*_s_, and Figure 9c illustrates that the bandwidth for CM suppression increases with increasing *l*_s_. Therefore, the bandwidth for CM suppression is primarily determined by the equivalent width of the SIW and the slot lengths.

According to Figure 5, Figure 6 and Figure 9, the bandwidth for CM suppression is usually wider than that for DM impedance matching and DM phase shift. Therefore, the operating bandwidth will be determined by the bandwidths for DM impedance matching and DM phase shift. In addition, we can find that *d*_s_ and *l*_f_ have obvious effects on the bandwidth for DM impedance matching but not on the bandwidths for DM phase shift and CM suppression. Therefore, the bandwidth for DM impedance matching can be controlled by *d*_s_ and *l*_f_. Furthermore, Δ*w*_s_ and Δ*l*_s_ have large effects on the bandwidth for DM phase shift. Thus, the bandwidth for DM phase shift can be primarily controlled by Δ*w*_s_ and Δ*l*_s_.

### 2.4. Design Procedure

The design procedure for the proposed balanced SIW phase shifter can be described as follows:

(1) Determine *a* according to the relationship of a, *f*_0_, and *w*_eff_ in Equations (1)–(3) to make the proposed design achieve the DM impedance matching and CM suppression at *f*_0_ simultaneously.
(2) Determine the initial values of *w*_s_, *d*_s_, *l*_s_, and *l*_f_ for the reference line according to the variation rules of the bandwidths for 15-dB DM impedance matching and 15-dB CM suppression in Figure 5 and Figure 9, respectively.
(3) Determine the initial values of *l*_d_ for different main lines according to the required phase shifts, and determine the initial values of *w*_s_, *d*_s_, *l*_s_, and *l*_f_ for different main lines according to the variation rules of the bandwidths for 15-dB DM impedance matching, DM phase shift, and 15-dB CM suppression in Figure 5, Figure 6 and Figure 9, respectively.
(4) Fine-tune the parameters in computer simulation technology (CST) to optimize the performance.

## 3. Results

Five prototypes of one reference line and four main lines are designed and fabricated at *f*_0_ = 3.5 GHz. According to the design procedure, the final dimensions are listed in Table 1. A four-port Agilent N5230C vector network analyser is used to test the proposed balanced SIW phase shifter.

**Figure 10:**
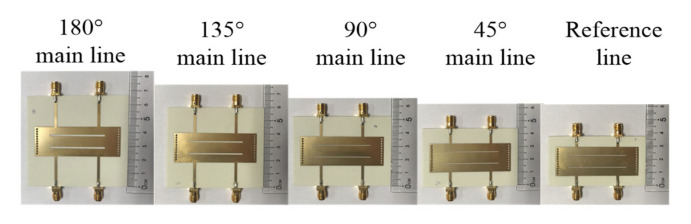
The photograph of the proposed balanced SIW phase shifter.

**Figure 11:**
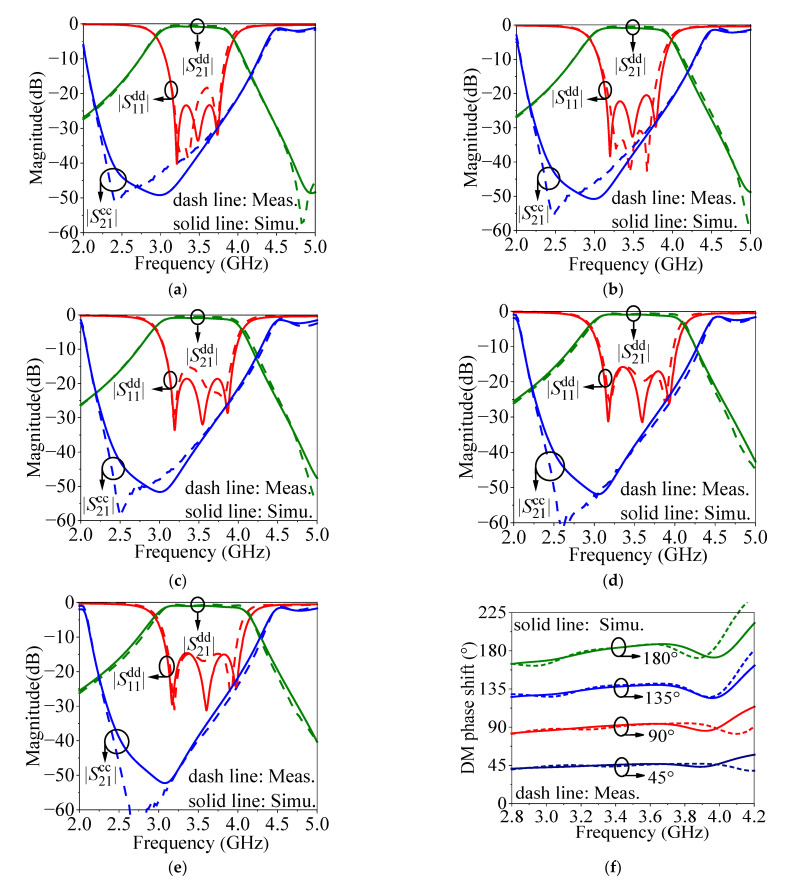
The simulated (Simu.) and measured (Meas.) results of the five prototypes. (**a**) *S*-parameters for the reference line. (**b**) *S*-parameters for the main line of 45° phase shifter. (**c**) *S*-parameters for the main line of 90° phase shifter. (**d**) *S*-parameters for the main line of 135° phase shifter. (**e**) *S*-parameters for the main line of 180° phase shifter. (**f**) DM phase shift.

The photograph and results of the five prototypes are shown in Figure 10 and Figure 11, respectively. For the proposed balanced SIW phase shifter, DM and CM *S*-parameters can be expressed as $S_{\mathrm{ij}}^{\mathrm{dd}}$ and $S_{\mathrm{ij}}^{\mathrm{cc}}$, respectively, where *i* and *j* are the port number, and *d* and *c* denote DM and CM, respectively.

It can be seen from Figure 11a–e that the reference line and main lines of 45°, 90°, 135°, and 180° phase shifters have the measured bandwidths for 15-dB DM return loss of 3.12 GHz to 3.82 GHz (20%), 3.12 GHz to 3.82 GHz (20%), 3.11 GHz to 3.91 GHz (22.9%), 3.12 GHz to 3.94 GHz (23.4%), and 3.12 GHz to 3.96 GHz (24%), with the bandwidth for 15-dB CM suppression of 2.07 GHz to 4.17 GHz (60%), 2.09 GHz to 4.16 GHz (59.1%), 2.12 GHz to 4.18 GHz (58.9%), 2.16 GHz to 4.22 GHz (58.9%), and 2.18 GHz to 4.22 GHz (58.3%), with the circuit size of λ_g_^2^, 1.2λ_g_^2^, 1.4λ_g_^2^, 1.6λ_g_^2^, and 1.8λ_g_^2^, with the minimum insertion losses of 0.38 dB, 0.41 dB, 0.5 dB, 0.58 dB, and 0.59 dB, respectively. It can be observed from Figure 11f that the bandwidths for the phase shifts of 45° ± 2°, 90° ± 4.5°, 135° ± 6°, and 180° ± 8° are 2.95 GHz to 4.07 GHz (32%), 2.9 GHz to 4.02 GHz (32%), 3.12 GHz to 3.88 GHz (21.7%), and 3.1 GHz to 3.85 GHz (21.4%), respectively. Therefore, the operating bandwidths for 45°, 90°, 135°, and 180° phase shifters are 20%, 20%, 20%, and 20%, respectively, which cover the 15-dB DM return loss, 15-dB CM suppression, and ± 5% phase deviation simultaneously.

Table 2 lists the demonstrated performances of the proposed design and other reported microstrip and SIW phase shifters. Compared to previously reported microstrip balanced phase shifters ([6,7,9]), the proposed SIW design has narrower bandwidth, but it would be more suitable for millimetre wave application. Compared to previously reported SIW phase shifters ([13,14,15,16,18,20,22]), the proposed design features a balanced topology with wideband CM suppression. In addition, compared to single-ended designs with equal lengths of the reference line and main lines from [13,15], the proposed design has a wider operating bandwidth. Compared to the wideband designs from [14,16], and the low insertion loss designs from [18,22], the size of the proposed design is more compact. Compared to the compact size design from [20], the proposed design has a larger phase shift range.

## 4. Conclusions

In this paper, a balanced phase shifter utilizing SIW with two slots is proposed, which has the features of wideband CM suppression, broad phase shift range, and an easily fabricated structure. DM and CM analysis, performance variation, and design procedure are performed to guide the practical design. The measurements of the final 45°, 90°, 135°, and 180° prototypes agree well with the theoretical prediction. The proposed balanced SIW phase shifter is believed to be capable of promoting the development of balanced microwave systems.

## Figures and Tables

**Table 1:** Dimensions of the proposed balanced SIW phase shifter (Unit: mm).

	Reference Line	45° Main Line	90° Main Line	135° Main Line	180° Main Line
*a*	59.1	59.1	59.1	59.1	59.1
*l*_f_	8.16	8.15	8.14	7.8	7.51
*s*	1.8	1.8	1.8	1.8	1.8
*d*	1.3	1.3	1.3	1.3	1.3
*g*	1.65	1.65	1.65	1.65	1.65
*d_s_*	8.36	8.2	8.2	7.8	7.56
*w_s_*	0.5	0.55	0.56	1	1.35
*l_s_*	41.6	41	40.4	38.9	38
*l_d_*	8	11.6	15.31	19.04	22.67
*w_p_*	1.83	1.83	1.83	1.83	1.83

**Table 2:** Performances comparison with previous works.

	*f*_0_ (GHz)	Phase Shift	FBW (%)	RL_min_ (dB)	IL_max_ (dB)	Size (λ_g_^2^)	15-dB CMS FBW (%)	n	Type
[6]	3.5	45° ± 2.3°	81	15	0.64	0.27	86	1	Microstrip, Balanced
		90° ± 4.7°	82		0.78	0.35			
[7]	3	180° ± 4.2°	73.8	14.87	0.96	0.8	75.2	1	Microstrip, Balanced
[9]	4	30.14° ± 2.2°	73	16	0.76	0.86	93.8	1	Microstrip, Balanced
		90.75° ± 4.1°	68.5	15	0.9	0.93	92.5		
		180.1° ± 5.5°	66.8	15	0.9	1.19	76		
[13]	24	15° ± 5°	10	10	1.5	4.51	N/A	1	SIW, Single-end
		30° ± 5°							
		60° ± 5°							
[14]	32	45° ± 3.5°	49	10.8	0.5	2.78	N/A	1	SIW, Single-end
		90° ± 2.5°	49	13	0.75	4.66			
[15]	10	11.25°	9.6	10	0.88	0.73	N/A	1	SIW, Single-end
		45°	6.9	10	2.32	0.73			
[16]	6	45° ± 2.5°	60	10	2.5	2.77	N/A	3	SIW, Single-end
		90° ± 3°	55			2.86			
[18]	33	43° ± 6°	42.4	12.5	0.52	5.43	N/A	3	SIW, Single-end
	34.4	90° ± 5°	12	10	0.52	15.2			
[20]	4.5	90° ± 3.5°	43	12	1.6	0.44	N/A	1	SIW, Single-end
[22]	41	90° ± 6°	39	25	0.2	3	N/A	1	SIW, Single-end
This work	3.5	45° ± 2°	20	15	0.8	1.2	59	1	SIW, Single-end
		90° ± 4.5°	20		0.82	1.4	59		
		135° ± 6°	20		0.93	1.6	58		
		180° ± 8°	20		1	1.8	57		

FBW: Fractional operating bandwidth; RL: Return loss; IL: Insertion loss; CMS: CM suppression; n: Number of layers.

## Data Availability

The data presented in this study are available on request from the corresponding author.

## References

[1] Zhu H., Sun H., Ding C., Guo Y.J. (2019). Wideband dual-polarized multiple beam-forming antenna arrays. IEEE Trans. Antennas Propag..

[2] Ren H., Li P., Gu Y., Arigong B. (2020). Phase shifter-relaxed and control-relaxed continuous steering multiple beamforming 4 × 4 butler matrix phased array. IEEE Trans. Circuits Syst. I Reg. Pap..

[3] Luo Q., Zhu X.-W., Yu C., Teng D.-D., Wang X., Chu C., Hong W., Zhu A. (2021). Linearization angle widened digital predistortion for 5G MIMO beamforming transmitters. IEEE Trans. Microw. Theory Tech..

[4] Zhang W., Shi J. (2019). A balanced phase shifter with common-mode suppression. IEEE Trans. Ind. Electron..

[5] Qiu L.-L., Zhu L. (2019). Balanced wideband phase shifters with good filtering property and common-mode suppression. IEEE Trans. Microw. Theory Tech..

[6] Zhang W., Xu K., Shi J., Shen Z.D. (2020). A compact single-layer balanced phase shifter with wide bandwidth and uniform reference line. IEEE Access.

[7] Alizadeh M.K., Shamsi H., Tavakoli M.B., Aliakbarian H. (2020). Simple ladder-like single-layer balanced wideband phase shifter with wide phase shift range and appropriate common-mode suppression. IET. Microw. Antennas Propag..

[8] Nie Y., Zhang W., Shi J. (2019). A compact balanced phase shifter with wideband common-mode suppression. IEEE Access.

[9] Qiu L.-L., Zhu L., Lyu Y.-P. (2019). Balanced wideband phase shifters with wide phase shift range and good common-mode suppression. IEEE Trans. Microw. Theory Tech..

[10] Shi J., Nie Y., Zhang W., Wu Y. (2021). Differential filtering phase shifter with wide common-mode suppression bandwidth and high frequency selectivity. IEEE Trans. Circuits Syst. II Exp. Briefs.

[11] Ding C., Meng F.-Y., Jin T., Lv J.-F., Mu H.-L., Wu Q. (2020). Tunable balanced liquid crystal phase shifter based on spoof surface plasmon polaritons with common-mode suppression. Liquid Cryst..

[12] Sellal K., Talbi L., Denidni T.A., Label J. (2008). Design and implementation of a substrate integrated waveguide phase shifter. IET Microw. Antennas Propag..

[13] Yang T., Ettorre M., Sauleau R. (2012). Novel phase shifter design based on substrate-integrated-waveguide technology. IEEE Microw. Wirel. Compon. Lett..

[14] Cheng Y.J., Hong W., Wu K. (2010). Broadband self-compensating phase shifter combining delay line and equal-length unequal-width phaser. IEEE Trans. Microw. Theroy Tech..

[15] Sellal K., Talbi L., Nedil M. (2012). Design and implementation of a controllable phase shifter using substrate integrated waveguide. IET Microw. Antennas Propag..

[16] Ebrahimpouri M., Nikmehr S., Pourziad A. (2014). Broadband compact SIW phase shifter using omega particles. IEEE Microw. Wirel. Compon. Lett..

[17] Liu S., Xu F. (2019). Novel substrate-integrated waveguide phase shifter and its application to six-port junction. IEEE Trans. Microw. Theroy Tech..

[18] Parment F., Ghiotto A., Vuong T.-P., Duchamp J.-M., Wu K. (2016). Double dielectric slab-loaded air-filled SIW phase shifters for high-performance millimeter-wave integration. IEEE Trans. Microw. Theroy Tech..

[19] Djerafi T., Wu K., Tatu S.O. (2015). Substrate-integrated waveguide phase shifter with rod-loaded artificial dielectric slab. Electron. Lett..

[20] Huang Y.M., Ding S., Wang G., Bozzi M. (2019). Compact equal-width equal-length phase shifter with slow-wave half-mode substrate integrated waveguide for 5G applications. IEEE Access.

[21] Zhang W., Shen Z., Xu K., Shi J. (2019). A compact wideband phase shifter using slotted substrate integrated waveguide. IEEE Microw. Wirel. Compon. Lett..

[22] Cano J.L., Villa E., Mediavilla A., Artal E. (2018). A wideband correlation and detection module based on substrate-integrated waveguide technology for radio astronomy applications. IEEE Trans. Microw. Theroy Tech..

[23] Ji Y., Ge L., Wang J., Chen Q., Wu W., Li Y. (2019). Reconfigurable phased-array antenna using continuously tunable substrate integrated waveguide phase shifter. IEEE Trans. Antennas Propag..

[24] Muneer B., Zhu Q., Xu S.J. (2015). A broadband tunable multilayer substrate integrated waveguide phase shifter. IEEE Microw. Wirel. Compon. Lett..

[25] Xu F., Wu K. (2005). Guide-wave and leakage characteristics of substrate integrated waveguide. IEEE Trans. Microw. Theroy Tech..

[26] Deslandes D., Wu K. (2003). Single-substrate integration technique of planar circuits and waveguide filters. IEEE Microw. Guid. Wave Lett..

[27] Oliner A.A. (1957). The impedance properties of narrow radiating slots in the broad face of rectangular waveguide. IEEE Trans. Antennas Propag..

